# Advances in Modeling the Inner Blood–Retinal Barrier: From Static Tissue Cell Cultures to Microphysiological Systems

**DOI:** 10.3390/ph18091374

**Published:** 2025-09-13

**Authors:** Aikaterini Apostolidi, Georgios Stergiopoulos, Sofia Bellou, Maria Markou, Theodore Fotsis, Carol Murphy, Eleni Bagli

**Affiliations:** 1 Foundation for Research & Technology-Hellas, Biomedical Research Institute (FORTH/BRI), 45110 Ioannina, Greece; apkater@gmail.com (A.A.); stergeo@yahoo.gr (G.S.); sofiabellou@uoi.gr (S.B.); mmarkou@bri.forth.gr (M.M.); thfotsis@uoi.gr (T.F.); carol_murphy@bri.forth.gr (C.M.); 2 Confocal Laser Scanning Microscopy Unit, Network of Research Supporting Laboratories (NRSL), University of Ioannina, 45110 Ioannina, Greece; 3 Laboratory of Biological Chemistry, Department of Medicine, School of Health Sciences, University of Ioannina, 45110 Ioannina, Greece

**Keywords:** inner blood–retinal barrier, cellular models, microphysiological systems, stem cells

## Abstract

**Background/Objectives:** The inner blood–retinal barrier (iBRB) is a specialized neurovascular interface essential for retinal homeostasis and visual function and is compromised in several vision-threating conditions. Therefore, the ability to model iBRB function and dysfunction in a controlled, reproducible and scalable manner is crucial for pharmaceutical research. However, the complex anatomy and physiology of the iBRB raise challenges for cell-based in vitro modeling. **Methods/Results**: This review follows the evolution of iBRB models—from simple monolayers of retinal endothelial cells (ECs) to sophisticated multicellular microphysiological systems (MPs). Advanced diverse microfluidic platforms aim to replicate key structural, biochemical and functional aspects of the iBRB, each incorporating distinct strategies regarding cell sourcing, device design, flow dynamics and functional readouts. **Conclusions**: Despite their limitations, these models are highly valuable for drug screening and mechanistic studies aimed at preserving or restoring barrier integrity while also helping to bridge the translational gap in ophthalmic drug discovery.

## 1. Introduction

### 1.1. The Inner Blood-Retinal Barrier (iBRB in Health and Disease)

**Figure 1:**
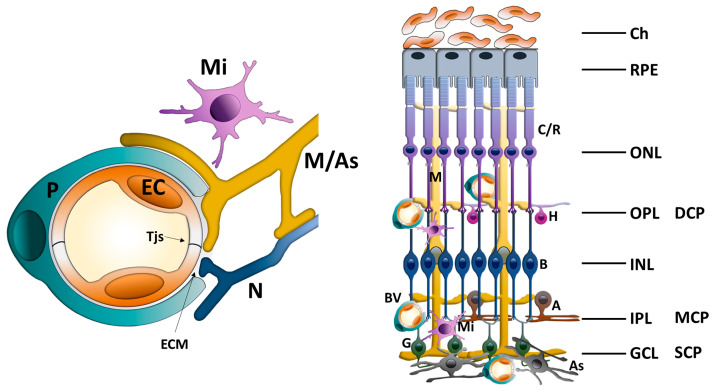
Schematic illustration of the iBRB as an integral part of the rNVU showing the interactions between ECs, pericytes, glial cells (astrocytes and Mϋller cells), retinal neural cells and microglia. iBRB: inner blood–retinal barrier; rNVU: retinal neurovascular unit; Tjs: tight junctions; ECM: extracellular matrix; P: pericyte; EC: endothelial cell; Mi: microglia; M: Mϋller glial cell; As: astrocyte; N: retinal neuron cell; C/R: cones/rods; H: horizontal cell; B: bipolar cell; A: amacrine; G: ganglion cell; BV: blood vessel; Ch: choroid; RPE: retina pigment epithelium; ONL: outer nuclear layer; OPL: outer plexiform layer; DCP: deep capillary plexus; INL: inner nuclear layer; IPL: inner plexiform layer; GCL: ganglion cell layer; MCP: middle capillary plexus; SCP: superficial capillary plexus.

The retina, as a direct extension of the central nervous system and functional continuation of the brain, has the highest oxygen demand per unit weight of any tissue in the body. In order to support this intense metabolic activity, its microenvironment requires highly controlled regulation. This is achieved through the blood–retinal barrier (BRB), which separates the retina from the systemic circulation to maintain homeostasis (Figure 1). The BRB plays a critical role in preserving the defined physiological conditions necessary for retinal function. Structurally, it consists of the following two components: the outer BRB (oBRB), formed by the tight junctions between retinal pigment epithelial (RPE) cells, and the iBRB, constituted by the tight junctions of retinal capillary endothelial cells (ECs) [1]. The outer retina, including the photoreceptor and RPE layers, receives its vascular supply from the choriocapillaris [2]. The choroidal endothelium is characterized by fenestrations and, subsequently, by increased endothelial permeability. Around 80% of the neuroretina’s oxygen and nutrient needs are met through passive diffusion from the choroidal blood supply [3]. In contrast, the inner two-thirds of the retina depend on the retinal capillary plexuses, whose non-fenestrated ECs form the iBRB. The iBRB is, therefore, a specialized vascular interface that regulates the exchange of nutrients, ions and metabolic waste between the blood and the neural retina.

#### 1.1.1. Beyond a Barrier: The iBRB as a Dynamic Component of the Neurovascular Unit (NVU)

With evolving insights into the dynamic signaling interactions between the retinal vasculature and surrounding neural components, the iBRB is increasingly viewed as part of a functional retinal NVU (rNVU) [4]. The integrated system involves various interacting cell types—including ECs, pericytes, astrocytes, Mϋller cells and microglia—that collectively contribute to the formation, maintenance and regulation of barrier integrity and retinal homeostasis [5] (Figure 1).

ECs form the core of the iBRB and similar to those of the blood–brain barrier (BBB), are highly specialized forming tight junctions (Tjs), adherens junctions and gap junctions [6]. The Tjs consist of transmembrane proteins, such as claudin-5, occludin and junctional adhesion molecules (JAMs-A/B/C) linked to intracellular scaffold proteins ZO-1, ZO-2 and ZO-3 [6]. Dynamic phosphorylation of TJ proteins controls paracellular permeability, while intracellular actin-myosin contractility modulates junctional tension [7]. Retinal ECs, unlike choroidal ECs, lack fenestrations and exhibit extremely low rates of transcytosis with few transcytotic vesicles, thereby regulating the selective transcellular transport [8,9]. In addition retinal ECs express low levels of vesicle transporters and high levels of efflux pumps (e.g., MDR1 and ABCG2), which together further control transcellular transport across the endothelium and contribute to the maintenance of a tightly regulated barrier [10]. The function of the EC junctions is finely tuned by interactions with surrounding rNVU components [4]. In this way, iBRB employs a multifaceted transport system [8]; Tjs and related junctional complexes primarily control paracellular flow, while energy-dependent vesicular pathways regulate transcellular movement. Key modulators, such as caveolin-1, MFSD2A and Wnt/β-catenin signaling, orchestrate the dynamic interplay among these routes, ensuring precise control of the retinal microenvironment and presenting, at the same time, possible therapeutic targets in retinal vascular diseases. This minimizes paracellular leakage, and the low rate of caveolae-mediated transcytosis, regulated by the expression of MFSD2A [11], is reflected in transendothelial electrical resistance (TEER) values of 1500–2000 Ω·cm^2^ in the healthy retina under physiological shear stress.

The basement membrane and the extracellular matrix (ECM) play key structural and signaling roles in the iBRB. Both ECs and pericytes are ensheathed by the basement membrane facilitating biochemical crosstalk and anchorage. It is composed of ECM proteins, such as collagen IV, nidogen, laminin and heparan sulfate proteoglycans, which support barrier integrity and influence cellular behavior [12]. Dynamic ECM remodeling can impact endothelial junctions and permeability, while integrin-mediated signaling from the ECM regulates survival, polarity and function of NVU cells [13].

Pericytes are embedded within the basement membrane and maintain close physical and functional contact with ECs. They are highly enriched in the retina compared to other vascular beds, with a near 1:1 ratio with ECs [14]. Pericytes regulate vascular stability, angiogenesis and barrier function via platelet-derived growth factor beta (PDGF-β)/PDGF receptor beta (PDGFRβ) signaling and other pathways like transforming growth factor beta (TGF-β) and angiopoietin 1 (Ang1)/Tie2 [15,16], while endothelial-derived PDGF-BB recruits pericytes during angiogenesis. Pericytes control capillary diameter through contractility and modulate endothelial permeability through the release of factors like angiopoietin [17]. They also deposit basement membrane proteins, including collagen IV α1/α2, laminin-511/521, nidogen and perlecan, creating a specialized ECM that supports barrier integrity [18]. Pericyte dysfunction or dropout, as seen in diabetic retinopathy (DR), leads to increased permeability and vascular instability [19].

Glial cells, notably Mϋller cells and astrocytes, provide metabolic and structural support. Astrocytes, predominantly located in the nerve fiber layer and ganglion cell layer, extend end-feet that ensheath vessels, influencing EC behavior through the secretion of Sonic hedgehog (Shh), cytokines and trophic factors [20,21,22]. They contribute to tight junction maintenance and ionic homeostasis, partly through calcium signaling and expression of aquaporin-4 and ion channels. While astrocytes are prominent regulators in the brain, their role in the retina appears more limited to the superficial vascular plexus (SVP) [20,23]. Mϋller cells are the principal macroglia in the retina and span all retinal layers. They intimately associate with vessels, especially in the intermediate and deep plexuses, and help regulate neurovascular coupling [24]. Mϋller cell end-feet ensheath capillaries, secreting neurotrophic factor (BDNF), which upregulates TJ proteins and promote antioxidant defenses. Their foot processes make contact with both neurons and vasculature, releasing gliotransmitters and vasoactive factors such as nitric oxide and prostaglandins [25,26]. In vivo and in vitro models have revealed the essential role of Mϋller cells in barrier maintenance, and their ablation leads to increased permeability [27].

Retinal neural cells are specialized neural cell types, including photoreceptors, bipolar cells, amacrine cells, horizontal cells and ganglion cells that together process visual information and maintain retinal function. Recent findings reveal that neural activity, particularly from starburst amacrine cells and cholinergic signaling, plays a crucial role in coordinating both angiogenesis and BRB formation in the retina [28]. Moreover, it has been shown that gaps in the Mϋller sheath, found mainly in the intermediate vascular plexus (IVP), permit diverse neurons, such as bipolar, amacrine and ganglion cells, to contact pericytes and ECs directly [29]. Although the precise nature of these interactions remains unclear due to the absence of presynaptic specializations, it is likely that neurons communicate with perivascular elements via non-synaptic pathways, such as gap junctions, transporter-mediated signaling or diffusible molecules like nitric oxide, while signals may also move in the opposite direction, from blood vessels to neurons and glia [30]. These findings underscore the cellular complexity and diversity of interactions within the NVU, highlighting retinal layer-specific contributions to the integrity of the iBRB [31].

Microglia are resident immune cells distributed throughout the synaptic layers of the retina and are closely associated with small vessels. Once thought to be primarily immune guardians, microglia are now recognized as active rNVU members that regulate vascular function through cytokine secretion, phagocytosis and interaction with neurons and pericytes [32,33,34]. Recent findings suggest that although microglia may not play a central role in maintaining iBRB integrity under normal physiological conditions, they become activated in the ischemic retina releasing pro-inflammatory cytokines like interleukin-1 beta (IL-1β) [35]. This, in turn, stimulates vascular endothelial growth factor (VEGF) secretion from Mϋller cells contributing to the breakdown of the iBRB [35]. Microglia proximity to metabolically active layers (inner and outer plexiform layers) and structural association with capillaries identifies them as potential regulators of blood flow, especially in the deep vascular plexus (DVP) and the SVP, where Mϋller cell regulation is limited [29].

In summary, while the iBRB refers to the unique features of the retinal ECs, the general make-up of the iBRB is an integral part of the rNVU [4], which is similar in structure and function to the brain NVU [36]. As a result, the iBRB relies on a complex interplay of multiple cellular components. ECs form the barrier core, pericytes provide structural and functional support, astrocytes and Mϋller cells regulate vascular behavior and barrier properties, and microglia contribute to immune surveillance and neurovascular signaling, while emerging research findings suggest that neural cells may contribute to rNVU function in a retinal layer-specific manner [29]. The ECM, particularly the basement membrane, is equally essential, serving as a physical scaffold and modulator of cellular communication. Impaired function of one or more of the involved cell types can lead to a wide range of retinal degenerative diseases, ultimately leading to blindness [4,8,10]. Therefore, understanding this multicellular and matrix coordination is crucial for accurate in vitro modeling of the iBRB and developing therapies targeting retinal vascular diseases.

#### 1.1.2. Breaking the Barrier: iBRB Dysfunction in the Retinal Disease

**Figure 2:**
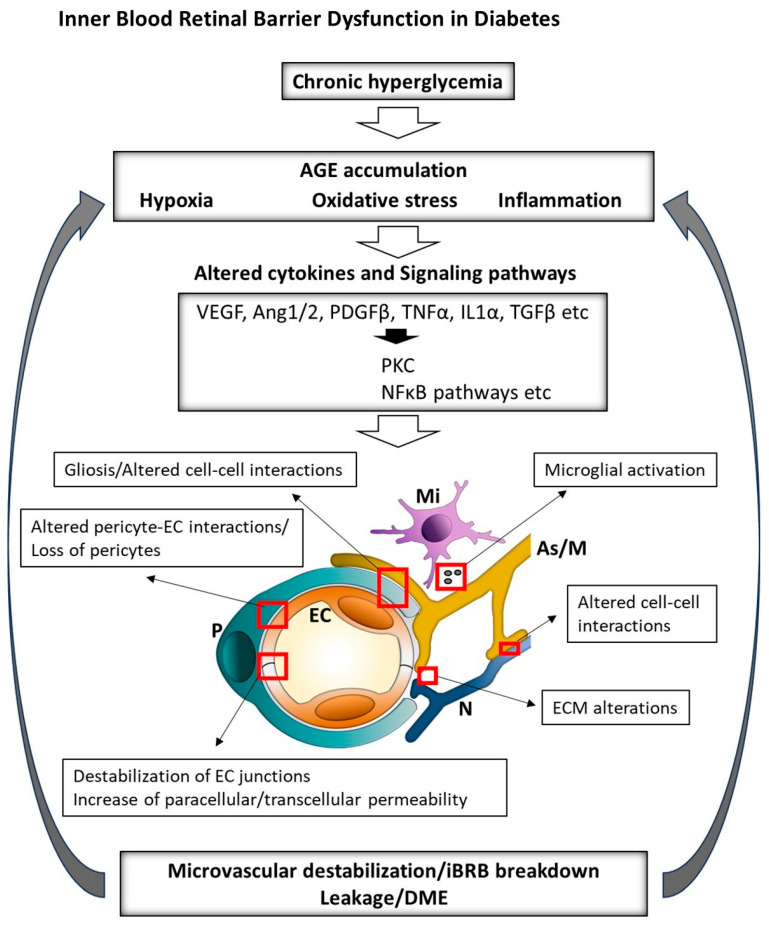
Overview of the key factors driving the pathogenesis of DME. Chronic hyperglycemia, hypoxia and inflammation alter cytokine expression and associated signaling pathways, leading to ECM damage, disruption of iBRB components and exacerbation of retinal hypoxia and inflammation. iBRB: inner blood–retinal barrier; ECM: extracellular matrix; DME: diabetic macular edema; AGEs: advanced glycation end-products; PKC: protein kinase C; VEGF: vascular endothelial growth factor; Ang: angiopoietin; PDGFβ: platelet-derived growth factor beta; TNFα: tumor necrosis factor alpha; IL1α: interleukin-1 alpha; TGFβ: transforming growth factor beta; NFκB: nuclear factor kappa-light-chain-enhancer of activated B cells; EC: endothelial cell; P: pericyte; As: astrocyte; M: Müller cell; Mi: microglia.

DR, one of the leading causes of blindness, affecting approximately 34% of diabetic patients worldwide [37,38,39,40,41], involves iBRB compromise [5]. Breakdown of the iBRB has also been implicated in retinal vein occlusion [42], retinopathy of prematurity [43], uveitis [44], retinoblastoma [45], Coat’s disease [46] and congenital vascular disorders linked to mutations in the Wnt/β-catenin signaling pathway, such as familial exudative vitreoretinopathy (FEVR) and Norrie disease [47,48,49]. In diabetic macular edema (DME), chronic hyperglycemia triggers oxidative stress, PKC activation and advanced glycation end product (AGE) accumulation, leading to TJ phosphorylation, occludin/VE-cadherin internalization and barrier leakage. Hypoxia-driven hypoxia-inducible factor-1 alpha (HIF-1α) stabilization elevates VEGF-A, activating VEGF receptor2 (VEGFR2)/Src signaling to disrupt junctions and enhance vesicular trafficking (52–54). Pro-inflammatory cytokines (tumor necrosis factor alpha (TNF-α), IL-1β and IL-6) further weaken the barrier via NF-κB signaling (54) and matrix metalloproteinase (MMP)-mediated basement membrane degradation. Mitochondrial fragmentation in ECs and pericytes, along with dysregulated choroidal flow, exacerbates dysfunction, manifesting clinically as macular fluid, cystoid changes and vision loss (55) (Figure 2). In age-related macular degeneration (AMD), affecting over 200 million people worldwide, complement activation and drusen deposition impair RPE, driving chronic inflammation, oxidative stress and secondary iBRB breakdown (57). However, the intricate intercellular dialogues among retinal cell populations under conditions of inflammation and oxidative stress remain poorly understood in the complex retinal milieu. Understanding these interactions highlights the need for advanced in vitro cellular platforms that recapitulate both normal and pathological rNVU states.

### 1.2. Rationale for In Vitro Modeling of the iBRB: Bridging the Translational Gap in Retinal Drug Development

The pharmaceutical industry faces a major challenge in translating early discovery findings into clinical success, especially in the field of retinal disease due to the complexity of iBRB physiology and pathology. Although anti-VEGF therapies have transformed DME and wet AMD treatment, 30–40% of patients exhibit incomplete or transient responses and frequent intravitreal injections carry risks of endophthalmitis and patient burden [50]. These challenges highlight the urgent need for identifying more precise therapeutic targets and using next-generation physiologically relevant cellular platforms for drug development and screening.

#### From Bench to Barrier: The Preclinical Challenge in Drug Development

Traditional cell-based assays utilize static monolayers of retinal microvascular ECs on transwell inserts, which facilitate high-throughput screening but lack the biomechanical cues of blood flow, ECM microenvironment and cellular crosstalk essential for accurate drug permeability, toxicity and metabolic evaluations. The development of physiologically relevant cellular models of the iBRB has also faced obstacles, such as the limited availability of primary retinal cells and the difficulty in preserving their phenotypic stability and proliferative capacity in vitro [51,52]. While immortalized cell lines offer convenience, they often lack the in vivo physiological characteristics of native cells and fail to replicate complex tissue-level interactions [53]. Finally, perfusion-free systems also fail to maintain stable barrier properties over extended periods of time, limiting chronic exposure studies relevant to DME and AMD. Consequently, these platforms show poor predictive accuracy. They are inadequate in understanding retinal physiology and retinal disease, as well as in developing therapeutic strategies, evaluating drug permeability, toxicity and efficacy under clinically relevant conditions.

Animal models—rodents, rabbits, and non-human primates—have offered valuable insights regarding our understanding of retinal vascular diseases and iBRB dysfunction. These models, including those with human disease gene mutations and targeted disruptions in key pathways such as VEGF and Wnt, helped to elucidate mechanisms of retinal angiogenesis and barrier breakdown (reviewed in [54]). However, they have limitations in translating to human conditions due to significant anatomical, molecular and behavioral differences, such as absence of a macula, differences in photoreceptor density and gene expression profiles [55,56,57]. Moreover, they exhibit species-specific differences in TJ protein expression (e.g., claudin-5 isoforms), pericyte coverage and transporter distribution, complicating translation of pharmacokinetic and pharmacodynamic data. As a result, disease manifestation and response to treatment are affected. Finally, while in vivo techniques enable the study of vascular leakage, they often fail to distinguish among specific transport mechanisms in order to reveal specific alterations of the iBRB in various retinal diseases [54]. Therefore, human-cell-based in vitro models offer a promising alternative by enabling more precise, high-throughput and physiologically relevant investigations of iBRB function and drug response while circumventing ethical issues, the expense of animal studies and variability inherent to animal models. Consequently, these translational shortcomings contribute to low success rates in drug development, with only 13.8% of drug candidates progressing from Phase I clinical trials to approval [58]. It is estimated that bringing a first-in-class drug to the market requires around ten years of development and an investment of approximately USD 2.5 billion [59].

Overall, despite significant advancements, it seems that the primary limitation in accurately replicating in vivo biological processes has been the lack of cellular complexity and native tissue architecture in traditional cell culture systems. Recent progress in stem cell engineering, along with the advent of omics technologies and organoid development, has enabled the creation of three-dimensional (3D) models derived from human embryonic stem cells (ESCs) and human-induced pluripotent stem cells (hiPSCs). HiPSCs have inexhaustible self-renewal and proliferation ability, can differentiate into all three germ layers and can be further directed into specialized cell types. To date, ophthalmic diseases represent a substantial share of hiPSC-based clinical trials, comprising up to 24.4% of all studies [60]. Organoids, 3D multicellular constructs, developed in vitro from ESCs or hiPSCs offer a more physiologically relevant platform by recapitulating the cellular diversity and complex architecture of native tissues, thereby enhancing the study of disease mechanisms at molecular, cellular and transcriptomic levels [61]. Specifically, in retinal research, stem-cell-derived retinal organoids (ROs) have opened new avenues for understanding human retinogenesis and generating patient-specific in vitro models, an essential step toward personalized medicine [62,63]. Often described as “miniature retinas”, ROs display a multilayered, laminated 3D architecture composed of key retinas cell types, including rods and cones, horizontal, bipolar, amacrine, ganglion and Mϋller cells, closely resembling the native neural retina, though they lack astrocytes, ECs and pericytes

The intrinsic complexity of these “mini-retinas” is being utilized by their integration with microphysiological organ-on-chip platforms, which can fine-tune their microenvironment and elevate their physiological relevance. Microphysiological organ-on-chip systems (MPs) are miniature devices which include tissue-specific cells, often in multicellular 3D arrangements, within a microfluidic environment that allows precise control of physical and biochemical conditions [64,65,66]. By replicating the architecture and function of native tissues, organ-on-chip models offer significant advantages over traditional 2D systems, including enhanced physiological relevance and customized microenvironments. Specifically, these advanced 3D cell culture platforms can effectively mimic complex vascular barriers such as the iBRB, oBRB and BBB [67,68,69,70]. Therefore, MPs are emerging as revolutionary platforms, offering human-based models that by integrating key elements of the native rNVU microenvironment, including multicellular ECM organization and perfusion, not only enhance physiological reliability but also enable high-throughput, reproducible drug screening and toxicity testing [70]. Therefore, the development and refinement of iBRB-specific MPs platforms utilizing ROs technology can reduce reliance on animal studies, improve safety and, ultimately, accelerate the delivery of novel therapeutics for retinal diseases, satisfying a pressing and growing public health need [71].

In this review, we focus on the current state and evolution of cellular models used to study the iBRB, with an emphasis on their relevance to drug development. While several reviews have discussed the BRB and its role in ocular health and disease, this work provides a distinct perspective by focusing specifically on the iBRB and its modeling. We highlight the progression from static single-cell and transwell-based co-culture systems to advanced MPs that better recapitulate the rNVU and iBRB, emphasizing how these evolving platforms address key translational challenges in drug discovery. Special emphasis is placed on how these models simulate barrier function, disease pathology and drug transport—underlining their potential to bridge the translational gap in ophthalmic drug discovery and screening. Despite recent advancements, critical challenges remain in retinal MPs development, such as standardization, throughput, cell sourcing and limited physiological relevance. While static mono- and multicellular iBRB models have provided valuable insights into barrier function and cell–cell interactions, they are also characterized by clear and quantifiable readouts that make them powerful tools for drug screening. This feature is particularly important as it informs the design of next-generation microphysiological platforms. Defining future directions—such as incorporating additional NVU components, employing advanced tissue-engineering strategies and integrating dynamic organ-on-chip technologies—is essential for achieving a more accurate representation of the iBRB.

## 2. Modeling the NVU of the Retina

### 2.1. Static Single-EC Culture Models: The Foundation

**Figure 3:**
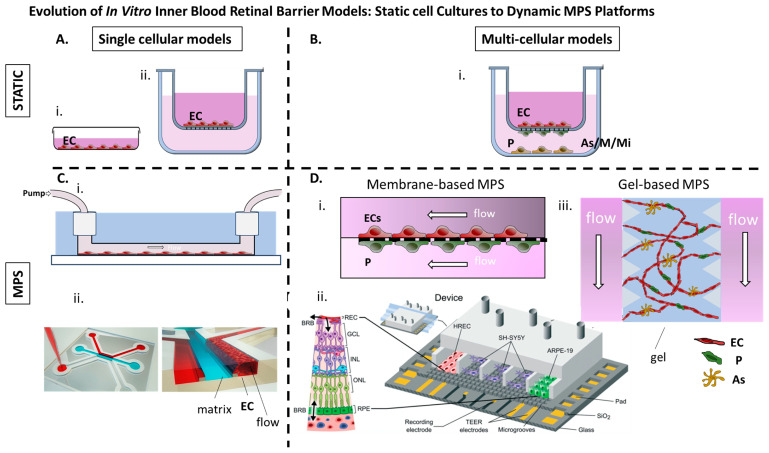
(**A**) i. Retinal ECs cultured as a monolayer on a dish. ii. Retinal ECs cultured on the apical side of a porous membrane (transwell insert). (**B**) i. Retinal ECs are cultured on the apical side of a porous membrane (transwell insert), while pericytes, glial cells, or microglia are cultured according to different setups on the basal side of the membrane or within the lower compartment. (**C**) i. Retinal ECs cultured on laminin coated microchannel under flow [72]. ii. Immortalized Retinal ECs cultured adjacent to a collagen I matrix under flow (OrganoPlate^®^ platform from MIMETAS) [73]. (**D**) i. MPs comprising two microchannels separated by a 10 μm microporous membrane, allowing co-culture of retinal microvascular ECs and immortalized human retinal pericytes on opposite sides to mimic the iBRB interface [71] ii. PDMS-based MPs with seven parallel compartments separated by microgrooves allowing for controlled communication between endothelial, epithelial, and neuronal cell types (Reproduced from [74]) with permission from the Royal Society of Chemistry) iii. Gel-based MPs in which human retinal microvascular ECs, pericytes, and astrocytes seeded in a fibrin hydrogel with flanking medium channels and allowed to be self-organized into perfusable microvascular networks [75]. BRB: Blood-retinal barrier, MPs: Microphysiological system, EC: Endothelial cells, P: Pericytes, As: Astrocytes, M: Mϋller cells, Mi: Microglia HREC: human retinal endothelial cells, GCL: ganglion cell layer, INL: inner nuclear layer ONL: outer nuclear layer RPE: retinal pigment epithtelium, TEER transendothelial electrical resistance.

Static single-cell culture models were the earliest in vitro approach for replicating the iBRB (Figure 3).

Among these, the TR-iBRB2 cell line—a conditionally immortalized rat retinal-capillary EC line—has been extensively used [76,77,78,79,80,81,82,83,84,85,86,87]. These cells express several essential iBRB transporter and tight junction proteins, including GLUT1, P-glycoprotein (ABCB1), LAT1, CRT, TauT, RFC1 and SR-BI, reflecting in vivo expression patterns and validating their use for transport and barrier function studies [88]. In addition, primary bovine and human retinal microvascular ECs, as well as hiPSC-derived retinal ECs, have been cultured on porous membranes or embedded within hydrogel inserts. These single-cell systems offer key advantages, such as simplicity, cost efficiency and compatibility with high-throughput screening. Standard functional assessments include TEER, which typically reaches quite low values of ~100–200 Ω·cm^2^, permeability assays with small- and large-molecular tracers and transporter activity assessed using radiolabeled or fluorescent substrates (Table 1).

The use of single-retinal-EC cultures enables the study of isolated endothelial-specific functions, particularly in transport kinetics and barrier regulation. TR-iBRB2 and similar lines retain transporter expression patterns and junctional features seen in vivo, allowing for a detailed mechanistic understanding. They are especially valuable for evaluating carrier-mediated drug delivery, understanding disease-associated transporter dysregulation and testing molecular interventions that target endothelial function (Table 1). However, they lack multicellular interactions and ECM complexity, leading to premature barrier breakdown and limited relevance for multi-factorial pathologies [103]. hRMVECs cultured on transwell inserts, for instance, showed low TEER values and were also insensitive to VEGFA treatment [73]. Finally, these single-retinal-EC models do not emulate flow-induced shear stress, which critically shapes EC morphology, alignment and barrier function through mechanosensitive pathways (e.g., PECAM-1, VE-cadherin and Notch signaling) [104]. Nonetheless, these models serve as valuable initial screens to identify compounds that modulate EC junctional integrity before further evaluation in complex platforms.

### 2.2. Static Multicellular Models: Toward Structural Complexity

The retina is composed of several specialized cell types, including retinal microvascular ECs, pericytes, astrocytes, Müller glia, microglia and neurons. Single-cell models—though useful—fail to account for the intricate cellular interactions that govern barrier function. Therefore, static multicellular co-culture and tri-culture systems have been developed using combinations of retinal cell types, often assembled in transwell systems to replicate the anatomical layering of the retina (Figure 2).

In these configurations, ECs are seeded on the apical side of a porous membrane, while pericytes, glial cells or microglia are cultured on the basal side or within the lower compartment. This setup not only separates the vascular and retinal compartments, mimicking physiological architecture, but also enables controlled paracrine signaling and direct cell–cell contact. When compared to monocultures, these co- and tri-culture models consistently show elevated TEER and reduced permeability, indicating improved barrier integrity. Adding pericytes and glia to static transwell enhanced NVU reliability. In co-culture setups, pericytes seeded on the basal side of a transwell membrane increased TEER by 2- to 3-fold [105]. Moreover, the highest TEER was observed in triple co-cultures, when glial cells (Mϋller or astrocytes) were added in the culture [53] (Table 2).

Basement membrane proteins coated on inserts, such as collagen IV and fibronectin, have been shown to support more robust junctional protein expression and long-term cell viability. As a result, these static multicellular models have allowed investigation of cell–cell signaling axes, such as EC-pericyte PDGF-BB/TGF-β loops and EC–glia VEGF/TNF-α responses, within a controlled environment, albeit without dynamic perfusion [112,114].

The assessment of the barrier in these multicellular static models includes basically TEER evaluation and permeability. While TEER primarily reflects paracellular barrier integrity, transwell transcytosis assays assess transcellular transport via clathrin- or caveolae-mediated pathways. In these assays, ECs alone or co-cultured with pericytes or astrocytes are grown on permeable membranes. Tracer molecules (e.g., radiolabeled sucrose, FITC-dextran, HRP or fluorescently tagged transferrin) are added to one compartment, and their movement across the monolayer is measured to evaluate active transport. Careful washing is essential to exclude paracellular leakage and isolate vesicular transport. These assays offer insights into the functional transcytotic capacity of the iBRB. In addition to TEER and permeability assays, immunofluorescence and qPCR have been employed to examine the expression of tight junction proteins (e.g., ZO-1, occludin and claudin-5) and key influx and efflux transporters (e.g., GLUT1 and P-gp) (Table 2). Such analyses confirm the enhanced morphological and functional features of the iBRB in multicellular systems.

Importantly, these models have been employed to simulate disease conditions relevant to drug development, including DR (via high glucose or AGEs), ischemia (via hypoxia) [35] and even viral infections (e.g., Ebola-like particles) [115]. Many of the observed responses, such as VEGF-mediated tight junction disruption or altered transporter expression, align closely with in vivo data, validating their relevance for translational research [111].

From a pharmaceutical perspective, these systems offer valuable platforms for evaluating drug permeability, toxicity and neurovascular protection. By using primary human retinal cells, interspecies variability can be reduced while improving the predictive value of preclinical studies. For instance, human retinal pericytes have been shown to respond differently than bovine pericytes under diabetic-like conditions, exhibiting increased susceptibility to apoptosis in response to fluctuating glucose levels [109,113]. Additionally, primary retinal ECs typically exhibit higher TEER and junctional protein expression than immortalized cell lines, though the latter are characterized by extended proliferative capacity and ease of use [53]. However, primary retinal cells are a limited resource because these cells can only be obtained from deceased donors, they can undergo replicative senescence, few cell lots are available from vendors, and donor information is limited, making it difficult to interpret factors that might affect their phenotypes [51,52]. Primary Müller cell cultures, as well as other primary retinal cell types, might also experience phenotypic instability during extended in vitro expansion. These changes challenge the use of primary Müller cells for long-term or high-throughput in vitro applications, especially in drug screening, as they may no longer represent the physiology of in vivo retinal glia [118]. HiPSC technology offers a promising solution to these challenges. HiPSC-derived microvascular ECs, astrocytes and retinal ganglion cells (RGCs) have been successfully incorporated into transwell models, demonstrating key barrier characteristics comparable to those observed in primary-cell-based systems [116].

In conclusion, static multicellular iBRB models are increasingly refined and have shown utility in drug development pipelines. They serve not only as tools for mechanistic investigation but also as scalable platforms for evaluating the safety, efficacy and delivery of retinal therapeutics in a controlled and physiologically relevant setting. However, caution must be taken in interpreting results from 2D static co-cultures. While they allow for complex signaling and junctional maturation, they lack the ECM components, 3D architecture and flow critical for replicating true vascular morphogenesis, such as lumen formation. These limitations highlight the need for further refinement of in vitro cellular models, including the integration of basement membrane components or transition to dynamic 3D systems.

### 2.3. Microphysiological Models of the iBRB: Bridging Physiology and Pharmacology

MPs constitute the current pinnacle of in vitro modeling for the iBRB, merging microfluidic design, advanced biomaterials and multicellular co-cultures to replicate the architecture and function of the rNVU under dynamic flow conditions. Transwell rNVU models—while capable of reaching TEER values on the order of 500 Ω·cm^2^ and suitable for acute permeability assays—fall short for studies requiring chronic exposure or physiologic shear. Fluid flow has been shown to assist the formation of tight endothelial or epithelial barriers [119]. Therefore, organ-on-chip platforms employing continuous perfusion have achieved TEER levels comparable to those measured in vivo and can maintain stable barrier function for a few weeks, thereby enabling longitudinal investigation of disease progression and pharmaceutical effects within a single device.

The following two main approaches have emerged: membrane-based microfluidic chips and gel-based self-assembled vascular networks (Table 3, Figure 2).

One of the earliest approaches involved the use of immortalized human retinal microvascular ECs (hTERT hRMVECs) cultured in a dual-lane OrganoPlate platform, which enabled the formation of tubular vascular structures adjacent to a collagen I matrix [73]. Notably, this model did not rely on a synthetic membrane but instead used ECM confinement and bidirectional perfusion to guide tubulogenesis and promote tight junction formation. Under flow conditions, endothelial monolayers exhibited markedly improved barrier function compared to static cultures, as evidenced by reduced permeability to 20 kDa and 70 kDa dextran tracers. The system proved responsive to cytokines, such as VEGFA and IL-1β, which increased permeability in a dose-dependent manner, thus validating the model for pharmacological screening of vascular-stabilizing agents. Automated imaging pipelines allowed for quantitative measurement of leakage and apparent permeability, making the platform particularly attractive for mid-throughput drug discovery. However, this model was limited by the absence of mural or supporting cells, such as pericytes and astrocytes, which are essential for the full replication of iBRB physiology.

To further increase accessibility and customization, another approach leveraged 3D printing technologies to fabricate molds for PDMS-based microfluidic devices, termed glial line (gLL) platforms [72]. These systems consisted of two reservoirs connected by microchannels of ~190 μm in hydraulic diameter, simulating the scale of retinal microenvironments. Devices were cast from molds created using fused deposition modeling (FDM) or stereolithography (SLA), with SLA yielding smoother and more consistent microchannel geometries. Flow was introduced using a syringe pump and cell behavior was assessed through live/dead staining, morphological quantification and cell shape index calculations. ECs tolerated shear stress better than neural cells, with increased elongation and structural alignment in flow conditions. Although the gLL system lacks vascular network formation and tight junction quantification, it serves as a valuable platform for studying basic flow-related cellular responses and for early-phase phenotypic screens.

A high-throughput alternative was presented with the PREDICT96 platform, a thermoplastic microfluidic system comprising 96 bilayer devices, each featuring two microchannels separated by a 10 μm microporous membrane [71]. This configuration enabled the co-culture of hRMVECs and immortalized human retinal pericytes on opposing sides of the membrane, supporting cell–cell communication and allowing for differential access to readouts, such as gene expression and cytokine secretion. The platform was designed to be compatible with scalable assays, including FITC-dextran permeability testing, qPCR and Luminex cytokine profiling. Notably, endothelial monolayers co-cultured with pericytes exhibited enhanced resistance to inflammatory stimulation, reduced IL-6 secretion and greater structural stability under flow. The capacity to monitor channel-specific responses significantly improved the interpretability of drug-screening data. Nevertheless, this model lacked the anatomical specificity and 3D architecture of the native iBRB, which may limit its predictive power for ophthalmic drug discovery.

A tri-culture strategy using a PDMS-based microfluidic device with seven parallel compartments separated by microgrooves has been also employed allowing for controlled communication between endothelial, epithelial and neuronal cell [74] types (Yeste et al., 2018). In this system, human retinal ECs, ARRPE-19 epithelial cells and SH-SY5Y neuroblastoma cells were cultured in discrete but interconnected compartments. The device incorporated platinum electrodes beneath the culture chambers to allow for real-time measurement of TEER, avoiding the need for complex electrode embedding. Fluorescent dextran assays, immunostaining for tight junction proteins such as ZO-1 and electrical impedance measurements confirmed the formation of functional barrier layers. This setup allowed for simultaneous assessment of multiple cell types and their contributions to barrier integrity, providing a multifaceted platform for analyzing drug-induced perturbations. However, direct heterotypic contact among cell populations was limited to paracrine signaling, the use of tumor-derived neuronal cells may not accurately reflect retinal neurophysiology and critical components of the iBRB such as pericytes and glial cells were missing.

The most comprehensive iBRB model to date was developed to specifically recapitulate the early pathological features of DR [75]. This platform combined primary hRMVECs, pericytes and astrocytes in a tri-culture system embedded in fibrin hydrogels. Under VEGF-guided conditions, the cells self-organized into perfusable microvascular networks with basement membrane deposition and functional junction formation. When subjected to chronic exposure to high glucose, TNF-α and IL-6, the system developed hallmark features of non-proliferative DR, including pericyte dropout, vascular regression, ghost vessel formation and increased expression of inflammatory cytokines. High-content imaging, RNA sequencing and cytokine profiling enable detailed analysis of disease mechanisms and identification of therapeutic targets. Targeted pharmacological interventions, such as inhibition of PDGFRβ, NOTCH and TIE2 signaling, produced phenotype-specific responses, demonstrating the utility of the platform for mechanism-based drug testing. This model provided insights into pericyte–endothelial interactions and mimicked aspects of basement membrane thickening, glycocalyx degradation and astrocyte loss observed in clinical DR. Although Müller cells, key cell mediators of iBRB, were not incorporated, the model offers a robust human-relevant system for translational drug development.

Collectively, these diverse microfluidic platforms reflect the growing sophistication of in vitro iBRB modeling. While some prioritize throughput and compatibility with standardized assays, others emphasize biological reliability through complex co-culture or hiPSC-derived systems. Perfusion plays a critical role in enhancing barrier integrity, yet flow strategies vary widely—from passive hydrostatic gradients [73] to actively pumped channels [71,72,75]. Similarly, strategies differ in their inclusion of supporting cell types, use of synthetic membranes versus self-assembled networks and degree of 3D architecture. From a pharmaceutical perspective, the choice of model depends on the intended application: high-throughput screens might benefit from robust bilayer systems, while mechanistic studies and precision medicine may favor patient-derived, biologically reliable platforms. Future directions include integration of additional rNVU components, continuous flow regimes and real-time biosensors for enhanced functional readouts. As these platforms continue to evolve, their impact on drug discovery and therapeutic development for retinal diseases is expected to grow rapidly.

## 3. Challenges & Future Directions

Modeling the iBRB has long been a challenge, evolving from single-cell culture systems to multicellular static platforms and, more recently, to MPs. Each of these approaches offers unique strengths and faces specific limitations (Table 4), but together they have provided critical insights into iBRB biology.

MPs, however, have transformed iBRB modeling approaches by recapitulating key aspects of retinal microarchitecture within perfusable, micrometer-scale devices. The implementation of MPs for ocular drug screening offers numerous advantages. By reducing dependence on animal models and incorporating human-specific NVU components, these systems enhance the physiological relevance of preclinical testing. Their capacity for high-content, phenotypic readouts allows for detailed assessments of drug effects, while the use of patient-derived cells opens the door to personalized medicine strategies. The rich datasets generated from microphysiological platforms can support pharmacokinetic and pharmacodynamic modeling, inform formulation strategies for both intravitreal and systemic delivery and enable early identification of off-target toxicities during the drug development process.

Yet, realizing their routine use in research and drug development hinges on overcoming several interrelated challenges as discussed below.

### 3.1. Key Areas for Addressing Current Challenges

#### 3.1.1. ECM

Maintaining a tight barrier requires a balance between cell–cell and cell–matrix adhesions and intracellular contractile forces transmitted via the cytoskeleton; disruption of this balance, such as on stiff substrates, can create paracellular gaps and increase permeability [120,121]. Therefore, the ECM plays a crucial role in supporting the structural organization and functional maturation of ECs and pericytes within MPs modeling the iBRB, as it provides the necessary biochemical signals and mechanical stiffness for tight junction formation and barrier integrity [122].

In iBRB-on-a-chip platforms, incorporating ECM components, such as collagen type IV, laminin, and fibronectin, helps recreate the native basement membrane, thereby enhancing cell adhesion, polarization, and intercellular communication among retinal microvascular cells. Moreover, ECM composition and architecture directly influence vascular morphogenesis and modulate the iBRB’s response to pathological stimuli, underscoring the importance of ECM optimization for accurate disease modeling and therapeutic screening [123].

Current challenges in selecting the appropriate ECM for MPs modeling the iBRB include accurately replicating the composition, mechanical properties and spatial organization of the native retinal microenvironment. The iBRB relies on a specialized basement membrane rich in collagen IV, nidogen, laminin and perlecan, yet many existing microphysiological platforms utilize simplified ECMs like collagen I or Matrigel, which may lack the biochemical cues needed for faithful EC and pericyte behavior [12]. Furthermore, batch variability and non-human origin of commonly used ECMs’ present issues for reproducibility and translational relevance [124].

Future directions include the use of tunable, defined ECM hydrogels that better mimic the stiffness and molecular composition of the retinal basement membrane. Advances in synthetic ECMs and decellularized tissue-derived matrices offer promise for greater control and biological relevance. Additionally, engineering ECMs to incorporate dynamic remodeling and cell-responsive degradation could better support long-term studies of iBRB function, maturation and disease progression, enabling more accurate modeling of pathological states like DR.

#### 3.1.2. Cellular Components

Equally critical is the establishment of well-characterized, renewable human cell sources. HiPSC-derived retinal ECs, pericytes and microglia offer an inexhaustible supply. In addition, hiPSC-derived ROs exhibit a multilayered organization recapitulating the 3D architecture and cellular complexity of the neural part of the human retina, comprising essential retinal cell types, such as Müller glia. Overall, it is possible to generate all cellular components of the rNVU from human hiPSCs, enabling the development of sophisticated multicellular models. However, several inherent limitations, such as biological variability among differentiation protocols and the variable maturation states of the derived cells, necessitate thorough benchmarking against primary retinal tissue [125]. Identifying tissue-specific markers of retinal ECs and pericytes is challenging due to limited human data and cell identity loss in culture [126]. Similarly, pericyte plasticity also complicates their characterization [127]. Recognition of tissue-specific markers is critical for validating retinal cells derived from iPSCs. RO heterogeneity, both across protocols and among individual organoids, is likely driven by epigenetic memory retained from the donor somatic cells, influencing lineage-specific differentiation of hiPSCs [128]. Moreover, differences in cell source and empirical differentiation methods contribute to inconsistent results between laboratories and experimental batches. Inadequate electrophysiological and omics-based characterization also hampers the functional validation of ROs as robust drug-screening tools.

Given the layer-specific diversity of the rNVU cellular components, such as astrocytes predominating in the SVP and Müller cells in the IVP, along with regional variation in neuronal populations interactions with the retinal vasculature [29], modeling layer-specific NVUs of the retina may be crucial for effective drug discovery and screening.

To address these issues, systematic improvements in differentiation protocols, tissue engineering, and co-culture are being pursued. Introducing ECs and microglia to ROs can enhance tissue maturity and mimic native retinal microenvironments. The beneficial synergy between ECs and stromal or mural cells in co-culture systems is well documented, leading to enhanced maturation [129]. Furthermore, evidence from oBRB studies demonstrated that co-culturing ECs with RPE, fibroblasts and pericytes enhances vascular specialization and cellular maturation. These cocultures promote choroidal fate in ECs, upregulate capillary and ECM markers and induce gene expression profiles that more closely resemble the native tissue. Importantly, the maturation benefits extend to supporting cells as well. Translating this strategy to iBRB modeling, co-culturing ECs with glial cells (e.g., astrocytes and Müller cells) and pericytes, all key regulators of iBRB, holds strong promise to promote a more physiologically relevant iBRB. Moreover, using technologies such as CRISPR/Cas9 to introduce reporter constructs, dynamic monitoring of growth factor expression and staged induction strategies can refine and standardize RO differentiation [130]. Multimodal pipelines that integrate single-cell RNA sequencing, mass-spectrometry-based proteomics and functional transporter assays are now being used validate differentiation protocols [131]. Together, these innovations aim to increase the maturity, reproducibility and functional reliability of the derived retinal cells, paving the way for their broader use in therapeutic discovery and disease modeling.

Furthermore, to facilitate broad distribution, advanced cryopreservation methods are being optimized to preserve post-thaw viability, barrier function and intercellular signaling competence, thereby enabling a multi-site banking approach for both academic and industrial laboratories.

#### 3.1.3. Reproducibility

Reproducibility remains a critical challenge for both stem-cell-derived models and organ-on-chip platforms. Variability in stem-cell differentiation efficiency, donor-to-donor heterogeneity, culture conditions and microfluidic device fabrication often leads to inconsistent outcomes (also mentioned above). The absence of a unified standard and quality-control framework, including parameters such as size, structural organization and biomarker expression, leads to variability in organoid quality across laboratories. To overcome these issues, strategies include the generation and following of guidelines in cell culture practice [132] and the adoption of fully defined, xeno-free media formulations to avoid variability associated with animal-derived supplements [133], as well as standardized protocols for iPSC differentiation with rigorous quality control checkpoints. Use of isogenic controls is also crucial, as this would allow researchers to isolate the specific effects of a gene mutation without the confounding influence of genetic background variables. In this context of iBRB and MPs studies, they provide a robust framework for distinguishing disease-related phenotypes from background noise, thereby enhancing the reliability of experimental outcomes. Incorporating tissue-engineering techniques that enable precise control over environmental factors could secure standardized production. Automation of culture handling and fluid delivery, combined with omics studies and integrated biosensors for continuous monitoring of TEER, oxygen, pH and cytokine secretion, could further enhance robustness while minimizing operator bias. In parallel, multi-institutional consortia, such as the IQ Consortium, the Organoids for Drug Screening initiative, and platforms like the MPs Development and Qualification Consortium, are driving the development of harmonized reporting standards, shared data repositories and open-source device designs [134,135]. These collaborative efforts collectively improve comparability and validation across laboratories, paving the way for more reproducible and translationally relevant iBRB models that can significantly advance mechanistic research and preclinical drug development.

#### 3.1.4. Monitoring Throughput

Real-time monitoring of culture parameters within retina-on-chip models provides both feedback for dynamic control and a non-invasive means to assess tissue integrity and specific responses, complementing endpoint analyses such as immunofluorescence microscopy. MPs developed in 3D gel-based systems, especially those involving mixed cell populations, can be technically challenging, low-throughput and difficult to image effectively. Quantifying permeability in 3D environments also presents significant obstacles. A 2D “flattened” MPs capillary model offers an alternative, simplifying the evaluation of endothelial barrier function and enabling more accessible high-throughput, imaging-based assays. However, imaging and assessment of such heterogeneous systems characterized by structural complexity and stratification of diverse cell types remain a major challenge. In addition, challenges, such as replicating physiological microenvironments managing drug diffusion and accurately analyzing multicellular drug responses, have limited broader adoption. Emerging technologies, such as microfluidics, bioprinting and fluorescence-based screening, are helping to improve drug testing within complex 3D cultures. Imaging advances like light-sheet and mainly lattice light-sheet microscopy now offer detailed 3D visualization of organoids, shedding light on cellular organization and guiding the optimization of culture conditions. Microfluidic systems with built-in sensors further enhance physiological relevance and allow for precise drug delivery and testing. For example, continuous TEER monitoring provides a reliable means to assess the integrity and maturation of vascular barriers in retina-on-chip models. Coupled with live imaging and other real-time readouts, this integrated approach enables non-invasive tracking and enhances the reliability of endpoint analyses in drug-screening studies.

#### 3.1.5. Scaling

Scaling MPs from PDMS prototypes to industrial-grade platforms under good manufacturing practice (GMP) requires both material innovation and process standardization. Injection molding of biocompatible thermoplastics, precision laser structuring of glass substrates and continuous roll-to-roll polymer film fabrication can deliver sub-micron pattering precision at scale [136]. High-throughput coating with ECM proteins and microtopographic patterning are now achievable via automated inkjet bioprinting and microcontact printing. To ensure consistency across large batches, non-destructive in-line quality-control techniques—optical coherence tomography for structural verification, Raman spectroscopy for biochemical assessment and contact angle measurements for surface wettability—have been implemented directly on production lines [137].

#### 3.1.6. Standardization and Accessibility

A key step toward widespread adoption of MPs, including iBRB models, is the harmonization of regulatory frameworks. Collaborative efforts across academia, industry and regulatory bodies are establishing standardized performance metrics—such as TEER thresholds, permeability assays and transporter activity test—along with transparent reposting practices to support their use in drug development submissions. Advances in automation and AI are increasing MPs throughput and reproducibility by enabling hands-free operation and predictive analytics for experimental optimization. Finally, economic feasibility and equitable access are being addressed through cost–benefit analyses, shared infrastructure models and partnerships that aim to make MPs platforms more assessable to smaller laboratories.

### 3.2. Conclusions

Looking ahead, the field of iBRB modeling must prioritize strategies to improve reproducibility, including the use of isogenic iPSC-derived cell lines, standardized ECM formulations and harmonized culture protocols. Incorporating additional retinal cell types, such as Müller glia, microglia and neurons, will further enhance physiological relevance and allow for modeling of complex disease mechanisms. The convergence of personalized MPs with immunocompetence and modular multi-organ integration promises to transform ophthalmic research and precision medicine. Patient-specific hiPSC-derived rNVU cells will enable individualized disease modeling and therapeutic screening. Advances in microfabrication, biosensors and automated analysis pipelines will be essential to enable high-throughput, standardized drug screening. Finally, closer collaboration between academia, industry and regulatory bodies is needed to establish shared benchmarks and qualification standards, ensuring that iBRB models can bridge the translational gap in ophthalmic drug discovery.

## Figures and Tables

**Table 1:** Static single-endothelial-cell culture models of the iBRB.

Study	Cell Type Used	Origin	Transporters/Trait Studied	Key Assays Performed	Notes/Disease Relevance
[76]	TR-iBRB2	Rat	Putrescine transport	Putrescine uptake	Mechanism of regulating retinal polyamine concentrations
[89]	TR-iBRB2	Rat	CAT1 (L-ornithine)	L-ornithine uptake	Hyperornithinemia and retinal degeneration
[90]	TR-iBRB2	Rat	Organic cation transporter	Verapamil uptake	Pharmacokinetics of cationic drugs
[88]	TR-iBRB2	Rat	OCTN2 (L-carnitine)	Acetyl-L-carnitine and L-carnitine uptake	Nutrient transport enhancement
[91]	TR-iBRB2	Rat	GLUT1 (dehydroascorbic acid)	Dehydroascorbic acid and ascorbic acid uptake	Oxidative stress model
[77]	TR-iBRB2	Rat	L-arginine transport under simvastatin/high glucose	L-arginine uptake	Diabetic condition modeling
[78]	TR-iBRB2	Rat	MCT (nicotinate)	Nicotinate uptake	Design of a suitable nicotinate dosage regimen
[79]	TR-iBRB2	Rat	Carrier-mediated transport systems (amantadine)	Transport assays	Application for retinal diseases such as glaucoma
[80]	TR-iBRB2	Rat	SMVT (Biotin)	Biotin uptake	Vitamin transport
[81]	TR-iBRB2	Rat	ATA2 system A transporter	Proline uptake	Amino acid neuroprotection
[82]	TR-iBRB2	Rat	GlyT1	Glycine uptake	Neurotransmission regulation
[83]	TR-iBRB2	Rat	RFC1 (MTF)	Folate uptake	Folate deficiency modeling
[84]	TR-iBRB2	Rat	ENT2 (adenosine)	Nucleoside uptake	Purinergic signaling
[92]	TR-iBRB2	Rat	MCT1 (lactic acid)	L-lactic acid uptake	Retinal metabolic acidosis
[85]	TR-iBRB2	Rat	CRT (creatine)	Creatine uptake	Energy homeostasis
[86]	TR-iBRB2	Rat	Organic cation/glucose-sensitive transporter	Paeonol uptake	Anti-inflammatory drug testing CNS delivery mechanisms
[87]	HRMECs	Rat	H+-gradient transporter (nicotine)	Nicotine uptake	CNS delivery mechanisms
[93]	BRMECs	Bovine	Junctional proteins	RT-PCR, Western blot, immunostaining	Molecular barrier integrity evaluation
[94]	HRMECs	Human	Adherens junctions	FITC-dextran flux assay, ESIC Immunocytochemistry	IL-33 role in endothelial permeability and iBRB integrity
[95]	HRMECs	Human	TRPV4 channel	Ca2+ imaging, impedance sensing, electrophysiology assessment	TRPV4 contribution to iBRB
[54]	HRMECs	Human	Transcytosis (clathrin-mediated, caveolae-mediated)	TEER, Transcytosis assay (Cy3-tagged transferrin, HRP)	Molecular regulators of EC permeability
[96]	HRMECs	Human	VEGF signaling modulation (endostatin)	TEER, permeability	Inflammation-induced barrier breakdown restoration
[97]	HRMECs	Human	Transendothelial permeability	FITC–dextran flux assay	Pathogenesis of Diabetic Retinopathy
[98]	HRMECs	Human	Vascular permeability	FITC-dextran flux assay xCELLigence electrical conductivity assays	Development of Diabetic Retinopathy
[99]	HRMECs	Human	Transendothelial permeability	TEER FITC-dextran flux assay Immunocytochemistry	Modeling of macular oedema
[83]	TR-iBRB2	Rat	RFC (MTF)	Folate uptake	Folate-related metabolic disorders
[100]	TR-iBRB2	Rat	TAUT	Taurine uptake	Neuroprotection function
[101]	TR-iBRB2	Rat	TAUT, GABA	GABA uptake	GABA transport properties
[82]	TR-iBRB2	Rat	GlyT1/system A	Glycine uptake	Neurotransmission and neuromodulation in the retina
[81]	TR-iBRB2	Rat	System A	L-proline uptake	concentration of small neutral amino acids
[102]	TR-iBRB2	Rat	L-cystine transporter, system x(-)(c)	L-cystine uptake	L-cystine transport mechanism

MCT: monocarboxylate transporters; SMVT: Na(+)-dependent multivitamin transporter; GlyT1: glycine transporter 1; RFC1: reduced folate carrier 1; MTF: methyltetrahydrofolate; ENT2: equilibrative nucleoside transporter 2; CRT: creatine transporter; BRMECs: bovine retinal microvascular endothelial cells; TRPV4: transient receptor potential isoform 4; HRP: horseradish peroxidase; CAT-1: cationic amino acid transporter 1; OCTN: organic cation/carnitine transporter; TAUT: taurine transporter; GABA: gamma-aminobutyric acid; ESIC: electric cell-substrate impedance sensing; HRMECs: human retinal microvascular endothelial cells.

**Table 2:** Static multicellular models of the iBRB.

Study	Cell Types Used	Transwell Setup	TEER Values & Comparison	Assays Performed	Pathological Conditions	In Vivo Comparison	Limitations
[106]	TR-iBRB2, TR-MUL5	TR-iBRB2 cells on the upper side of a rat tail collagen type I-coated cell culture insert (pore size: 3.0 μm) cultured with TR-MUL5 seeded on the backside membrane	Not measured	Gene expression (microarray, RT-qPCR), ALP activity	Direct and paracrine signaling	PAI-1/Id2 modulation supported known angiogenic control	Lacked barrier assays
[107]	hRMECs, BRPCs	hRMECs on transwell membranes cultured individually with BRPCs on the bottom side of the filter or on the bottom of the well	Not stated	Proliferation, apoptosis, cord formation	EC–mural cell interaction	Demonstrated vessel formation and Notch signaling	Method-oriented; no quantitative barrier validation
[108]	ECs, Bovine, Pericytes	Direct, indirect and 3D collagen co-culture	Not stated	Migration, proliferation, tube formation	Pericyte recruitment mechanisms	Modeled mural cell support role	Qualitative; lacked disease modeling
[109]	hRPs, hRECs	hRECs on PET transwell membranes (1 µm pore size) cultured individually or with hRPs on the bottom side of the membrane	Not stated	IHC for TJ proteins	High glucose		Limited to endothelial–pericyte interaction
[110]	HRMECs, BV2 & primary rat microglia	HRMECs on the bottom side of fibronectin coated membrane cultured with BV2 cells seeded on the apical side of the transwell membrane.	Not stated	Confocal imaging phagocytosis assay	Diabetic retinopathy	Mimicked microglial engulfment observed in vivo	Limited to microglia–endothelial interaction
[111]	Rat RMEC, rat RMGC	RMEC on gelatin coated insert co-cultured with RMGC on the bottom side of the insert	TEER dropped dose-dependently with AGEs	TEER, VEGF/PEDF concentration (ELISA)	AGE-induced BRB dysfunction	VEGF/PEDF modulation consistent with in vivo DR	Rat models; limited temporal scale
[112]	HUVECs, rat Müller glia, rat astrocytes	HUVECs on collagen IV/fibronectin membrane (0.4 μm pore size) cultured individually or with rat Müller glia on the bottom side of the insert’s membrane (combo culture). Conditioned media (CM) of the individual cell type cultures were taken	Combo cultures had lowest TEER under hyperglycemia	TEER, ROS production	Hyperglycemia with AGEs	In vitro responses mirrored known in vivo phenotypes	Used HUVECs instead of retinal ECs; limited chronic modeling
[113]	HMEC, HRPs, MIO-M1	HMEC on transwell insert cultured with HRP on the bottom side of the insert and MIO-M1 on the bottom of the well	TEER values not provided	Permeability, transporter expression, viability	Diabetic retinopathy, thiamine deficiency	Inferred transporter dynamics mirrored in vivo	Non-retinal HMEC; cell lines used
[53]	Primary BRECs, BRPCs, rat astrocytes	BRECs on collagen IV coated polycarbonate insert (pore size: 0.4 mm) cultured alone or with BRPCs/rat astrocytes on the bottom side of the insert or on the bottom of the well (a total of 5 setups)	BRECs + BRPCs + Rat astrocytes > BRECs + Rat astrocytes > BRECs * the highest TEER values in triple co-cultures with astrocytes at the bottom side of the filter and BRPCs at the bottom of the well	TEER, permeability (FITC-dextran), transporter expression	VEGF-induced DME	VEGF effects on GLUT1, P-gp consistent with in vivo	Mixed species
[105]	Rat RECs and RMPs	RECs on polyethylene terephthalate membrane (pore size: 0.4 μm), cultured alone or with RMPs on the bottom side of the membrane (direct contact-DC) or with RMPs on the bottom of the well (indirect contact-IDC)	RECs + RMPs (DC) > RECs > RECs + RMPs (IDC)	TEER, MMP assays, FITC-Na permeability WB of TJ proteins	Loss of direct contacts of pericytes with ECs in diabetic retinopathy	Reflected MMP-2/9 effect in vivo	Rat origin; only pericyte influence explored
[35]	bEnd.3, QMMuC01, BV2	bEnd.3 on insert (pore size: 0.4 μm), cultured alone or with QMMuC-1 on the bottom side of the insert and BV2 on the bottom of the well	bEnd.3 + QMMuC-1 + BV2 = * bEnd.3 + QMMuC-1 > bEnd.3 * normoxic conditions	TEER, EB assay, FITC-Na permeability IHC and RT-PCR for TJ proteins	Hypoxia-induced BRB breakdown	Modeled hypoxia-mediated VEGF upregulation	Immortalized cell lines; mouse origin; lacks pericytes
[114]	Rat RCECs, rat RMCs	RCECs on collagen IV/fibronectin-coated transwell polycarbonate filter (pore size: 0.4 μm) cultured alone or with RMCs on the bottom side of the filter	RMCs + RCECs > RCECs	TEER, TGF-β/Smad activation	Diabetic conditions, high glucose, ACE	Supported known pathways of barrier breakdown	Rat origin; moderate model duration low TEER values
[115]	Immortalized HRECs, HRPs, primary HRAs	HRECs on collagen IV coated insert (pore size: 0.4 μm) cultured alone or with HRPs on the bottom side of the insert’s membrane and HRAs on the bottom of the well	HRECs + HRPs + HRAs > HRECs	TEER, Na-F permeability, IHC for TJ proteins	Ebola virus exposure	Confirmed breakdown mechanism in vivo	Immortalized cells, limited chronic modeling, low TEER values
[116]	Human iPSC-derived MVECs, astrocytes, RGCs	MVECs on collagen IV/fibronectin-coated insert, cultured alone or with astrocytes and RGCs on the bottom of the well	MVEC + astrocytes + RGCs > MVEC	TEER, Na-F permeability (Na-F), P-gp function (Rhodamine 123) IHC for TJ proteins	Glaucoma-associated barrier dysfunction	Recapitulated key barrier traits similar to primary models	Brain-like MVECs used; lacks direct cell–cell contact
[117]	rRMECs rMG	rMG, rRMECs on collagen IV/fibronectin coated polyester (PET) membrane with a 10 μm thickness and 0.4 μm pore size individually or in COMBOs (rRMECs on the top and rMG on the bottom side of the insert’s membrane)	COMBOs > rRMECs > rMG	TEER, TNF-α challenge, anti-VEGF-A treatment	Hyperglycemia, inflammation, anti-VEGF-A response	TEER patterns upon treatment match in vivo data	Rat origin; required validation in long-term and 3D studies

RT-qPCR: reverse transcription quantitative real-time PCR; ALP: alkaline phosphatase; PAI-1: plasminogen activator inhibitor-1; rRMECs: rat retinal microvascular endothelial cells; rMG: rat primary Müller glia; P-gp: P-glycoprotein; MVECs: microvascular endothelial cells; RGCs: retinal ganglion cells; iPSC: induced pluripotent stem cells; HRPs: human retinal pericytes; HRAs: human retinal astrocytes; HRECs: human retinal endothelial cells; RCECs: retinal capillary endothelial cells; rMCs: rat retinal Müller cells; bEnd.3: immortalized mouse brain microvascular EC line; BV2: Immortalized Mouse Microglial cell line; QMMuC-1: Queen’s University Murine Müller glia Clone-1 (immortalized mouse retina MCs); PET: polyethylene terephthalate; EB: Evans Blue; FITC: fluorescein-5-isothiocyanate; RT-PCR: reverse transcriptase polymerase chain reaction; IHC: immunocytochemistry; RMECs: retinal microvascular endothelial cells; RMGCs: retinal Müller glial cells; ELISA: enzyme-linked immunosorbent assay; AGEs: advanced glycosylation end-products; VEGF: vascular endothelial growth factor; RMPs: retinal microvascular pericytes; PEDF: pigment epithelial derivative factor; BRECs: bovine retinal endothelial cells; BRPCs: Primary Bovine Retinal Pericytes, RA: Rat Astrocytes, HMECs: human microvascular endothelial cells; MIO-M1: human Müller cell line;; HRMECs: human retinal microvascular endothelial cells; BRPs: bovine retinal pericytes; TR-iBRB2: immortalized rat retinal capillary endothelial cell line; TR-MUL5: Müller cell line; GLUT1: glucose transporter protein type 1; TNF-α: tumor necrosis factor alpha; ROS: reactive oxygen species; MMP: matrix metalloproteinase; TEER: trans-endothelial electrical resistance; Tjs: tight junctions; TGF-β: transforming growth factor beta.

**Table 3:** Microphysiological models of the iBRB.

Study/Platform	Cell Types	Strategy	Readouts	Disease Model	High Throughput/Screening Potential
[73] (OrganoPlate)	hTERT-hRMVECs	Gel-based (collagen I); no membrane	Fluorescent dextran permeability; automated imaging; leakage score; apparent permeability	Retinal barrier leakage (e.g., VEGFA)	Moderate to high (automated imaging)
[71] (PREDICT96)	hMVECs + pericytes	Bilayer with microporous membrane	FITC-dextran permeability; cytokine profiling (Luminex); qPCR; image-based screening	Barrier disruption & inflammation	High (96-well format, automation ready)
[74] (Microgroove-TEER chip)	HRECs + ARPE-19 + SH-SY5Y	Microgroove-separated compartments	TEER (integrated electrodes); fluorescent tracer permeability; ZO-1 immunostaining; confocal imaging	iBRB mimic; neurovascular interface	Moderate (multi-modal but complex setup)
[72] (3D-printed gLL)	Rat RECs + rat RNCs (r28)	Channel-based PDMS device (FDM/SLA molds)	Cell viability (live/dead); morphology (CSI); bead-tracked flow validation	Shear stress effects; retinal cells	Low (proof-of-concept, not scalable)
[75] (Tri-culture fibrin MVN)	Primary HRMVECs + pericytes + astrocytes	Fibrin gel-based 3D vascular network	Confocal imaging; TRITC-dextran permeability; perfusability; RNA-seq; Luminex; apoptosis (caspase-3/7, LDH-Glo CytotoxicityAssay)	Diabetic retinopathy (NPDR model)	Moderate (complex but highly informative)

hRMVECs: human retinal microvascular endothelial cells; VEGFA: vascular endothelial factor A; TEER: trans-endothelial electrical resistance; hMVECs: human retinal microvascular endothelial cells; HRECs: human retinal endothelial cellss; ZO-1: zonula occludens protein 1; iBRB: inner blood–retinal barrier; NPDR: non-proliferative diabetic retinopathy; RECs: retinal capillary endothelial cells; RNCs: retinal neural cells; gLL: glial line; PDMS: polydimethylsiloxane; FDM/SLA: fused deposition modeling/stereolithography; MVNs: microvascular networks.

**Table 4:** Comparative summary of iBRB models, outlining their main characteristics, advantages and limitations, with emphasis on their applicability for mechanistic studies and drug discovery.

Strategy	Advantages	Limitations
**Single-cell cultures** (e.g., retinal endothelial cells)	- Clear simple readouts (TEER, permeability assays) - High reproducibility - Cost-effective and scalable for drug screening	- Lack of physiological complexity - Absence of pericytes, astrocytes and ECM - Limited translational relevance
**Static co-culture models** (e.g., Transwell systems with endothelial cells plus pericytes/glial cells)	- Better mimics cell–cell interactions - More accurate barrier properties (compared to single-cell systems) - Well-established and widely used in drug permeability assays	- 2D and static, lacking shear stress and flow - Limited ability to recapitulate 3D tissue organization - Moderate reproducibility across laboratories
**Microphysiological systems (MPs)** (organ-on-chip, 3D models)	- Closely mimic in vivo microenvironment - Incorporate flow, shear stress and ECM - Potential real-time monitoring (TEER, imaging) - High translational potential for drug testing and disease modeling	- Technically complex and costly - Reproducibility issues between devices and laboratories - Distinct design configurations are required depending on the application (no standardized setup can yet accommodate all drug development needs) - Standardization and quality control still under development

## Data Availability

No new data were created or analyzed in this study.
